# Keypoint regression strategy and angle loss based YOLO for object detection

**DOI:** 10.1038/s41598-023-47398-w

**Published:** 2023-11-17

**Authors:** Xiuling Wang, Lingkun Kong, Zhiguo Zhang, Haixia Wang, Xiao Lu

**Affiliations:** 1 College of Electrical Engineering and Automation, Shandong University of Science and Technology, Qingdao, 266590 China; 2 College of Energy Storage Technology, Shandong University of Science and Technology, Qingdao, 266590 China

**Keywords:** Computer science, Information technology

## Abstract

The YOLOv4 approach has gained significant popularity in industrial object detection due to its impressive real-time processing speed and relatively favorable accuracy. However, it has been observed that YOLOv4 faces challenges in accurately detecting small objects. Its bounding box regression strategy is rigid and fails to effectively leverage the asymmetric characteristics of objects, limiting its ability to enhance object detection accuracy. This paper proposes an enhanced version of YOLOv4 called KR–AL–YOLO (keypoint regression strategy and angle loss based YOLOv4). The KR–AL–YOLO approach introduces two customized modules: an keypoint regression strategy and an angle-loss function. These modules contribute to improving the algorithm’s detection accuracy by enabling more precise localization of objects. Additionally, KR–AL–YOLO adopts an improved feature fusion technique, which facilitates enhanced information flow within the network, thereby further enhancing accuracy performance. Experimental evaluations conducted on the COCO2017 dataset demonstrate the effectiveness of the proposed method. KR–AL–YOLO achieves an average precision of 45.6%, surpassing both YOLOv4 and certain previously developed one-stage detectors. The utilization of keypoint regression strategy and the incorporation of robust feature fusion contribute to superior object detection accuracy in KR–AL–YOLO compared to YOLOv4.

## Introduction

Object detection remains a challenging research area within computer vision, encompassing the localization and classification of objects within a scene. The successful advancements in computer vision have significantly improved the accuracy of object detection, which plays a pivotal role in various applications. Traditional approaches such as V–J detector^1^, histogram of oriented gradient (HOG)^2^, local binary patterns (LBP)^3^, and deformable parts models (DPM)^4^ have demonstrated good results in terms of accuracy and speed by relying on handcrafted object features like edges, key points, or templates. However, the limited diversity of these manual design elements makes it challenging to meet the high-precision requirements of complex object detection tasks with diverse target properties.

In recent years, object detection methods based on convolutional neural networks (CNNs) have gained significant attention and outperformed traditional approaches by leveraging features automatically extracted from CNNs instead of handcrafted features. The breakthrough of CNNs came with AlexNet^5^ in 2012, which ignited interest in neural networks and deep learning, propelling them to the forefront of artificial intelligence research. Subsequent approaches, such as OverFeat^6^ and region-based CNN (R-CNN)^7^, introduced AlexNet-based categorization, positioning detection, and refined object proposal techniques based on CNNs, respectively. These developments led to deep learning-based object detection methods becoming a prominent research area. Fast R-CNN^8^ and Faster R-CNN^9^ were subsequently introduced to enhance the speed of the R-CNN series methods by employing selective search and the regional proposal network (RPN)^10^ instead of sliding window search, simplifying region proposal generation and improving object detection time.

Typically, object detection networks based on the sliding window or RPN frameworks consist of two stages: candidate-region generation and classification. However, this two-stage process often hinders real-time performance due to its inherent low speed. The You Only Look Once (YOLO)^11^ method, a representative one-stage approach, addresses this issue by treating object identification as a regression problem, generating prediction boundary boxes and categories simultaneously by inputting an image into a neural network. Unlike two-stage methods, YOLO directly trains a single convolutional network for candidate generation and end-to-end classification, resulting in real-time object detection performance. Subsequently, other one-stage approaches, such as single-shot detection (SSD)^12^ and RetinaNet^13^, were proposed, further improving the speed and accuracy of object detection.

Among various one-stage detectors, YOLO, a classical one-stage object detection algorithm, has gained popularity in industrial detection due to its remarkable speed and high accuracy. The YOLO application has evolved over time, with YOLOv2 (YOLO9000)^14^ and YOLOv3^15^ significantly enhancing object detection accuracy and speed compared to the original YOLO approach. Several enhancement techniques based on YOLO have continually improved its effectiveness and have also found applications in various domains, such as UAV-based traffic monitoring^16^. These approaches have achieved favorable outcomes by enhancing YOLO from different perspectives.

YOLOv4^17^ further improved the backbone network structure and introduced feature fusion mechanisms to enhance interaction with target features, resulting in an efficient and accurate model with high mean average precision (mAP). However, the YOLOv4 algorithm’s bounding box prediction strategy, which predicts the center coordinates, width, and height of each box, has limitations when dealing with objects whose most distinctive features are not centered within the bounding box. For targets with significant asymmetric characteristics, the rigid bounding box prediction method fails to fully exploit the object’s asymmetric properties, thereby limiting detection accuracy improvement.

To address this limitation, we propose an object detection approach called KR–AL–YOLO (keypoint regression and angle loss based YOLOv4). KR–AL–YOLO optimizes the bounding box regression of the network model by introducing an keypoint regression (KR) strategy. Additionally, a novel angle loss (AL) function is designed to adapt to the new regression strategy, effectively updating the network model parameters and enhancing algorithm accuracy. Improved feature fusion techniques are also introduced to reinforce the original feature fusion in YOLOv4.

With the progression of time, numerous improved versions of the YOLO series algorithms have been proposed, such as YOLOv5^18^, YOLOv6^19^, YOLOv7^20^, and YOLOv8^21^. These methods have shown significant advancements in both accuracy and speed for object detection compared to YOLOv4. For instance, YOLOv5^18^ refined the network architecture based on YOLOv4, replacing the activation function with Leaky ReLU, transitioning from CBM to CBL structures, and incorporating a focus module to enhance the receptive field during the convolution process. YOLOv6^19^ focused on lightweighting the model for industrial applications, resulting in improved speed. YOLOv7^20^ made substantial improvements in model structure (CSP to ELAN), convolutional strategies (Conv to RepConv), and label assignment methods, leading to notable performance enhancements. YOLOv8^21^ drew inspiration from YOLOv7’s ELAN structure, substituted the C3 structure with C2f, replaced the Head section with a decoupled head structure. The regression approach also shifted from anchor-based to anchor-free, and the loss computation introduced the TaskAlignedAssigner positive sample allocation strategy. These advancements collectively position YOLOv8 as one of the most cutting-edge methods available. However, YOLOv4 remains widely utilized in practical projects. Thus, our objective revolves around validating and incorporating these proposed techniques within the YOLOv4 framework.

In this paper, we present the KR–AL–YOLO approach, which improves upon YOLOv4 by introducing the keypoint regression strategy instead of center point regression. The model is trained to regress prediction boxes in a more flexible format, leveraging the asymmetry between left and right features as well as upper and lower features of the target objects. We regard all the locations inside the object bounding box as positives with a keypoint and four offset to detect objects. We design a native angle-loss function to refine the network model parameters, further improving algorithm accuracy. Furthermore, condidering the effectiveness of BiFPN feature fusion in handling diverse object scales and its successful integration into state-of-the-art object detection models, we introduce this enhanced feature fusion methods to bolster YOLOv4’s capabilities. The proposed KR–AL–YOLO approach achieves higher mAP than YOLOv4 while maintaining real-time detection speed.

The main contributions of this paper are as follows:

1. We introduce KR–AL–YOLO, a novel approach within the YOLOv4 framework that incorporates a keypoint regression strategy and an angle loss mechanism. By capitalizing on the inherent asymmetry of object features, our model excels at regressing prediction boxes in a more adaptable output format, enhancing its object localization capabilities.
2. We develop an original angle-loss function that effectively updates the model’s network parameters. This innovative loss function contributes significantly to refining the algorithm’s precision, thereby leading to improved detection accuracy.
3. We introduce and integrate enhanced feature fusion (BiFPN) techniques into the YOLOv4 architecture. These techniques serve to bolster the model’s overall performance by augmenting its ability to extract meaningful object representations. Our proposed KR–AL–YOLO method seamlessly incorporates these advancements, resulting in a substantial enhancement over the baseline YOLOv4 model’s detection capabilities.

The rest of this paper is organized as follows: “Related Works” section provides a brief review of related works, “Methods” section explains the proposed approaches in detail, “Experiments” section presents experimental results and discussion, and finally, “Conclusions” section concludes the paper.

## Related works

Object detection has witnessed significant advancements, with two general categories of CNN-based approaches: anchor-based and anchor-free methods. In this section, we provide an overview of these approaches.

### Anchor-based object detectors

Anchor-based detectors utilize predefined anchor boxes of various sizes to predict bounding boxes. Girshick et al.^7^ introduced R-CNN in 2014, which was the first object detection method to leverage CNNs for feature extraction. It employed CNNs to perform convolutional operations on candidate regions and updated weights using gradient descent for automatic feature selection. However, the method suffered from a large number of redundant candidate boxes, impacting both speed and accuracy. To address this, He et al.^22^ proposed SPPNet, a spatial pyramid pooling network that shared computation across multiple regions within the CNN forward pass, thereby accelerating R-CNN. Subsequent methods such as Fast R-CNN^8^ and Faster R-CNN^9^ introduced selective search and the Region Proposal Network (RPN), respectively, to simplify region proposal generation and enhance detection speed. Liu et al.^12^ proposed SSD, which achieved good performance for small objects by independently detecting objects on multiple feature maps.

The YOLO algorithm and its variants, including YOLOv2^14^, YOLOv3^15^, YOLOv4^17^ and other YOLO-based variants^23,24^, have gained significant popularity. YOLOv2 removed the constraint that a grid cell can only anticipate a single object and predicted offsets of the desired center point relative to the grid. YOLOv3 introduced a multi-scale fusion feature pyramid network (FPN), while YOLOv4 optimized calculation efficiency by incorporating mosaic data enhancement and improved feature fusion using techniques such as SPP and PANet. Furthermore, certain YOLO-based approaches, such as RSOD^16^, have innovated upon the YOLO methodology from distinct perspectives. These methods have been applied in diverse domains and have yielded promising outcomes. These advancements in the YOLO series methods have made them popular choices for one-stage object detection due to their state-of-the-art accuracy and real-time performance.

### Anchor-free object detectors

Anchor-free methods typically consider the object’s center point or pre-defined/self-learned keypoints to define positive samples and predict bounding boxes. YOLOv1, for instance, divides an image into grids and uses the grid cell containing the object’s center to predict the bounding box. DenseBox^25^ focuses on a filled circle at the object’s center for identification and directly predicts the bounding box. CornerNet^26^ predicts the top-left and bottom-right corners of an object, defining the bounding box based on these keypoints. However, distinguishing corners for dense objects becomes challenging. CenterNet^27^ addresses this by employing cascade corner pooling and center pooling to collect information from the corners and central regions, respectively, resulting in improved precision and recall. FCOS^28^ analyzes the differences between anchor-based and anchor-free detectors and introduces an adaptive training sample selection (ATSS) to bridge this gap. Zhang et al.^29^ propose a learning-to-match (LTM) method that enables flexible anchor matching by formulating detector training within the maximum likelihood estimation framework, extending the anchor-free paradigm. Additionally, transformer-based approaches, such as DETR^30^ and ViDT^31^, have been introduced in object detection, simplifying the pipeline by directly predicting sets of objects and eliminating non-maximum suppression and anchor generation steps. The ViDT approach introduces the Reconconfigured Attention Module, enhancing feature extraction capabilities. It also employs a lightweight neck-free architecture to reduce computational costs. Additionally, it introduces a novel concept of token matching for knowledge distillation, leading to improved model performance. This makes ViDT one of the top-performing methods in object detection currently.

## Methods

This paper propose an enhanced object detection approach called KR–AL–YOLO. Our method builds upon YOLOv4 by introducing a flexible bounding box regression strategy, specifically keypoint regression. We also propose a novel angle-loss function and an improved fusion method for integrating multiscale features of objects. The KR–AL–YOLO model is constructed and trained to achieve accurate object detection.

The original YOLOv4 network predicts four coordinates for each bounding box and converts them to the coordinates of the center point, width, and height using a predefined formula. However, objects often possess uneven features, and their most distinctive characteristics may not align with the geometric center. The standard bounding box regression approach, which assumes equal distances from the center to the boundaries, may not be suitable for objects with asymmetric features. Instead of using the geometric center of the bounding box, we redefine the center point of the network prediction as arbitrary keypoint within the feature map.

### YOLOv4 approach

We selected YOLOv4 as our baseline due to its simplicity and high performance. YOLOv4 utilizes CSPDarkNet53 as the backbone network, PANet as the feature fusion network, and YOLO head for prediction. It employs three feature maps of different scales to detect objects.

**Figure 1:**
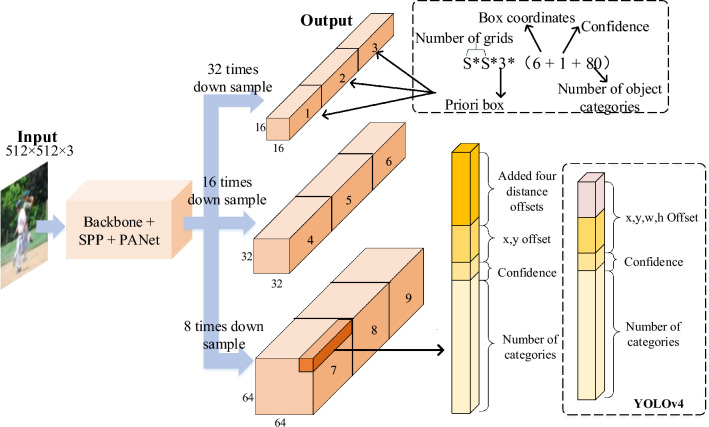
Network architecture of KR–AL–YOLO. We propose a simple yet effective bounding box regression strategy to make the regression flexible. We followed the reported default network parameters for the backbone. All the numbers are computed with $512\times 512$ input.

The input image of size $512 \times 512$ is processed by the CSPDarkNet53 network to extract image features. The SPP (Spatial Pyramid Pooling) module enhances the receptive field for each position in the feature map. The PANet feature fusion network combines top–down transmission of semantic features and bottom-up transmission of robust localization features. Parameter aggregation is performed from different backbone networks for various detector levels. As shown in Fig. 1, the input image goes through several convolutional layers to obtain feature maps at three scales with downsampling factors of 32, 16, and 8, respectively. Lower-resolution feature maps with larger receptive fields are responsible for detecting large objects, while higher-resolution feature maps with smaller receptive fields are suitable for detecting smaller targets. YOLO divides the image into an $S \times S$ grid, and each grid cell predicts the target if the center point of the object falls within it. Three anchor boxes are assigned to each grid, characterized by 85 dimensions. These dimensions include the center (*x*, *y*) of the box relative to the grid cell, width and height (*w*, *h*) relative to the entire image, confidence of the prediction, and 80 object classes from the COCO dataset. The confidence indicates the likelihood that the predicted box contains an object.

### Keypoint regression strategy

The YOLO model generates predictions for target dimensions in a format of (4 + 1 + 80), where 4, 1, and 80 represent the offsets of the predicted box center point coordinate, the width and height offsets of the predicted object, the confidence of the prediction, and the number of object categories in the COCO dataset, respectively. These four predicted values are then transformed into the target’s center point coordinate, width, and height using the YOLO position prediction decoding formula.

In YOLOv4’s bounding box regression, the distances from the center point to the left and right boundaries, as well as to the upper and lower boundaries of the bounding box, are assumed to be equal. However, this rigid regression method fails to account for objects with prominent asymmetric characteristics, resulting in low Intersection over Union (IoU) scores between predictions and ground truth.

**Figure 2:**
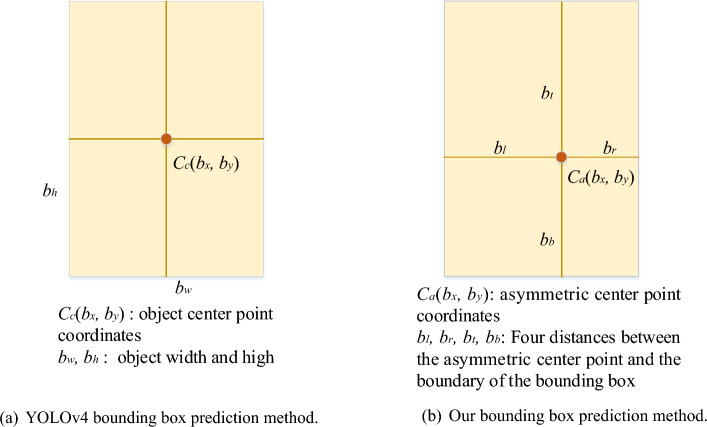
Simple schematic diagram of bounding box prediction methods.

Moreover, the most significant features of an object may not necessarily align with the geometric center of the feature map. Instead, the center of gravity within the feature map may better capture the feature distribution. Considering the gravity center as a crucial keypoint for refining predictions, we propose a straightforward yet effective strategy called keypoint regression to introduce flexibility into the regression process. We regard all the locations inside the object bounding box as positives with a keypoint and four offset to detect objects. As depicted in Fig. 2a and b, we replace the four values of the bounding box center point offset ($t_x$ and $t_y$) and bounding box size offset ($t_w$ and $t_h$) with six values: keypoint information ($t^a_{x}$ and $t^a_{y}$) and the distances from the keypoint to each boundary of the bounding box ($t_l, t_t, t_r$, and $t_b$).

Following YOLOv4’s approach, we detect objects of various sizes on different levels of feature maps. Specifically, we utilize three levels of feature maps generated by the CSPDarkNet53 backbone, followed by upsampling and CBL (convolution, normalization, and leaky rectified linear unit) modules. Consequently, the network predicts six coordinates for each bounding box at each scale. These six values are converted into the final prediction output ($b_{x}, b_{y}, b_l, b_t, b_r$, and $b_b$) using the following equations:

1 $$\begin{aligned}{} & {} {\left\{ \begin{array}{ll} b_x&{} = \varvec{\sigma }(t^a_x) + c_x,\\ b_y&{} = \varvec{\sigma }(t^a_y) + c_y,\\ b_l&{} = p_{w}e^{t_l},\\ b_r&{} = p_{w}e^{t_r},\\ b_t&{} = p_{h}e^{t_t},\\ b_b&{} = p_{h}e^{t_b}, \end{array}\right. } \end{aligned}$$

2 $$\begin{aligned}{} & {} \varvec{\sigma }(x)=\frac{1}{1+e^{-x}} , \end{aligned}$$

where $\varvec{\sigma }$ represents the sigmoid function in Eq. (2), $c_{x}$ and $c_{y}$ denote the coordinates of the top-left corner of the grid responsible for predicting the object, and $p_{w}$ and $p_{h}$ correspond to the width and height of the corresponding anchor relative to the output layer, respectively.

The network output dimension at each scale changes from $N\times N\times 3\times 85$ to $N\times N\times 3\times 87$. The minimum and maximum values are $b_x-b_l$, $b_x+b_r$, and $b_y-b_t$, $b_y+b_b$ in the *x* and *y* directions, respectively. These six coordinates determine the prediction box and enable the generation of high-quality bounding boxes, thus improving recall and precision.

### Angle loss

**Figure 3:**
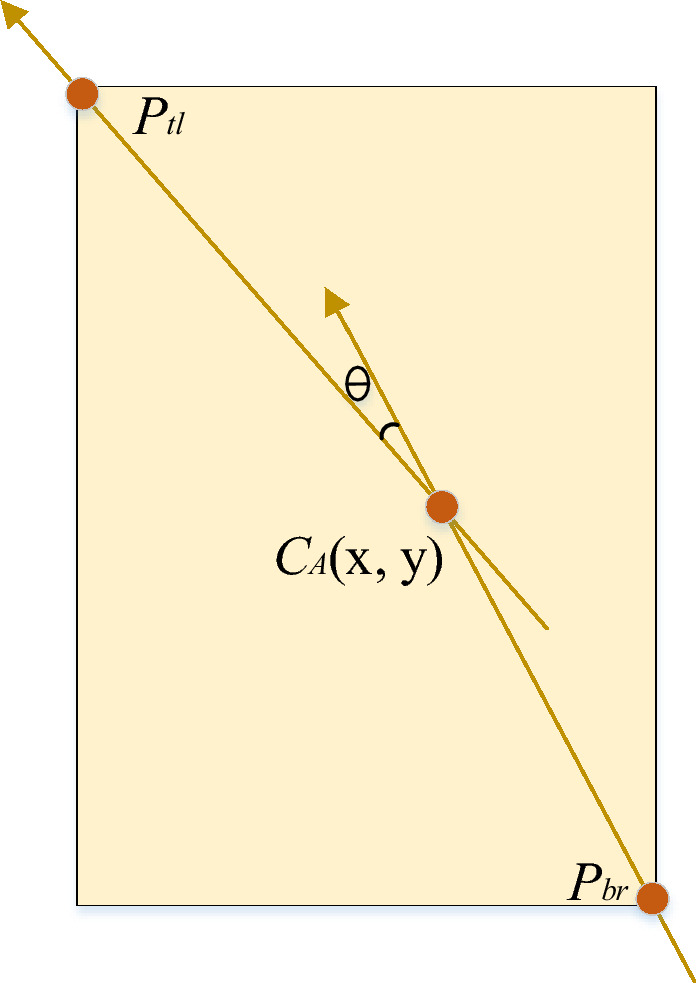
Angle formation diagram.

The keypoint regression method refines the localization of predicted bounding boxes by incorporating the keypoint location and the distances from the keypoint to each boundary. These coordinated parameters collectively determine the positions of the predictions. In cases where $b_l \ne b_r$ or $b_t \ne b_b$, the keypoint and the center of the bounding box do not coincide, resulting in an angle between the vectors originating from the top-left corners and the bottom-right corner towards the keypoint (Fig. 3). Motivated by cosine similarity, this study introduces a novel angle-loss function that is included in the overall loss computation and backpropagation process. The total adopted loss function is defined as:

3 $$\begin{aligned} L = L_{loc}+L_{conf}+L_{prob}+L_{angle}, \end{aligned}$$

where $L_{loc}$, $L_{conf}$, and $L_{prob}$ represent the complete IoU regression loss, confidence loss, and multi-class cross-entropy classification loss, respectively. These loss components correspond to those utilized in YOLOv4.

$L_{angle}$ denotes the proposed angle loss, which measures the cosine of the angle between the two vectors $\varvec{\mu } = \overrightarrow{{C_A}{P_{tl}}}$ and $\varvec{\nu } = \overrightarrow{{P_{br}}{C_A}}$, where $C_A$, $P_{tl}$, and $P_{br}$ represent the keypoint, top-left, and bottom-right points, respectively (Fig. 3). The angle loss values range from 0 to 1, with 1 indicating that the vectors are pointing in the same direction and 0 indicating orthogonality (i.e., perpendicularity) between the vectors. Thus, the angle loss is formulated as follows:

4 $$\begin{aligned} L_{angle} = 1/N*\sum \limits _{i = 0}^{N}(s * (\cos \hat{\theta }_i -\cos \theta _i))^2, \end{aligned}$$

where *N* denotes the batch size, $\theta$ represents the angle between the two vectors $\varvec{\mu }$ and $\varvec{\nu }$, $\hat{\theta }_i$ denotes the ground truth angle for the i-th sample, ${\theta }_i$ denotes the predicted angle for the i-th sample, and *s* represents a scaling factor that controls the magnitude of the logits. The angle $\theta$ is calculated based on the six coordinates $b_x, b_y, b_l, b_t, b_r$, and $b_b$, and its value range is $[-90, 90]$:

5 $$\begin{aligned} \begin{aligned} \cos \theta&= (\varvec{\mu }*\varvec{\nu })/(|\varvec{\mu }||\varvec{\nu }|)\\&=(\mu _x \mu _y+\nu _x \nu _y)/\left( \sqrt{{\mu _x}^2+{\mu _y}^2}\sqrt{{\nu _x}^2+{\nu _x}^2}\right) , \end{aligned} \end{aligned}$$

where $\mu _x = -b_l$ and $\mu _y = -b_t$ denote the *x* and *y* coordinates of $\varvec{\mu }$, while $\nu _x = b_r$ and $\nu _y = b_b$ denote the *x* and *y* coordinates of $\varvec{\nu }$, respectively.

### BiFPN feature fusion

In the context of object detection tasks, the integration of low-level physical features with high-level semantic features is commonly employed through skip connections or hypercolumns to enhance overall performance. High-level features capture abstract object semantics, while low-level features provide more detailed object descriptions. However, the presence of a substantial semantic gap between different levels often fails to provide robust feature support for multiscale visual recognition tasks. The objective of the neck component in object detection algorithms is to aggregate target feature information as comprehensively as possible. This aggregation occurs prior to feeding the object feature information, extracted from the backbone network, back into the detection head. This approach aims to mitigate the loss of fine-grained information when the feature information of small objects at lower levels interacts with semantic information at higher levels of abstraction. The feature fusion method employed in YOLOv4 is PANet. FPN (Feature Pyramid Network) is an efficient feature fusion technique that propagates high-level semantic features from top to bottom, thereby strengthening the entire feature pyramid. However, this unidirectional information flow solely enhances semantic information and does not transmit positional information. As FPN transmits shallow features from the bottom to the top, the shallow feature information becomes attenuated due to the numerous convolutional layers applied in reaching the topmost level. To address this issue, PANet introduces an additional path that facilitates the bidirectional transmission of low-level localization features, enabling pairwise aggregation of parameters.

**Figure 4:**
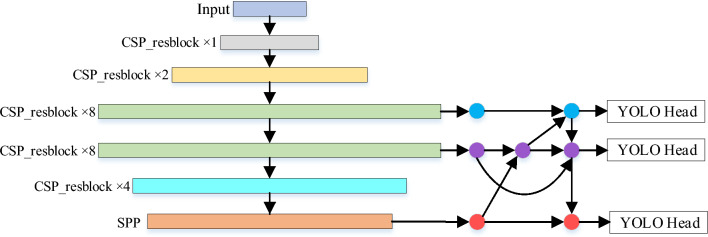
Network structure diagram after changing the YOLOv4 feature fusion mode to the BiFPN.

This study adopts the bidirectional Feature Pyramid Network (BiFPN) fusion method^32^ to enhance the YOLOv4 algorithm and achieve a more comprehensive fusion of features extracted from the backbone network. Figure 4 illustrates the modified neck section of YOLOv4. Compared to PANet, BiFPN offers superior feature fusion capabilities and efficient cross-scale connections. BiFPN streamlines the bidirectional network by eliminating nodes with only one input edge, as they contribute less to the overall feature network. Furthermore, BiFPN achieves enhanced feature fusion by introducing an additional edge from the original input to the output node, repeating the bidirectional path multiple times at the same layer. These improvements lead to effective feature fusion without significant increases in computational cost. We have employed BiFPN as a connection mechanism between nodes to facilitate improved outcomes in YOLOv4.

## Experiments

### Experimental setup and implementation

#### Datasets

To assess the effectiveness of the KR–AL–YOLO approach in comparison to other object detection methods, particularly YOLOv4, we conducted experiments on the COCO dataset. The COCO2017 dataset, utilized for the object detection experiments, comprised over 100,000 images encompassing 80 object categories. Specifically, the COCO training dataset, consisting of 11,726 images, was employed for model training, while the COCO validation dataset was utilized to evaluate the performance of KR–AL–YOLO.

#### Model training

The experiments were conducted on a GTX Titan V100 12G GPU with CUDA 10.0 and cuDNN 7. The implementation was carried out using the PyTorch deep learning framework within the Python programming language, utilizing the PyCharm platform. We adopted the default network parameters and the number of iterations specified in the literature as the baseline for our results. Our approach introduces regression strategies, angle-loss functions, and BiFPN feature fusion techniques while keeping the original backbone intact, all of which are lightweight computational methods. These methods hardly impose any additional computational burden during both training and inference processes. As a result, our model’s training and inference times remain nearly identical to those of the original YOLOv4 model.

Consistent with other object detection approaches like YOLOv4, we set $IoU_{pred}^{truth}$ to 0.5. The initial learning rate was set to 0.01, and a cosine-annealing decay learning rate schedule was employed for adjustments. The training batch size was set to 12, and the total number of iterations was 300 epochs.

### Experiments on the COCO dataset

**Table 1:** Comparison of improvements on each component in the proposed method on COCO test-val2017.

Method	$AP_{50:95}$	$AP_{50}$	$AP_{S}$	$AP_M$	$AP_L$
YOLOv4	45.4	64.9	23.0	50.7	**62.7**
+KR	45.5	65.3	25.4	50.9	61.6
+KR+AL	45.4	65.3	**26.2**	50.7	61.6
+KR+BiFPN	45.5	65.4	25.4	50.9	62.2
+KR+AL+BiFPN	**45.6**	**65.4**	25.4	**51.0**	62.0

’+KR’ represents the YOLOv4 with keypoint regression strategy. ’+KR+AL’ represents the YOLOv4 with keypoint regression strategy and angle loss.

**Table 2:** Results on the COCO test-val2017.

Method	Average precision (%)						
	Size	$AP_{50}$	$AP_{50:95}$	$AP_{75}$	$AP_S$	$AP_M$	$AP_L$
RetinaNet^13^	500	53.1	34.4	36.8	14.7	38.5	49.1
SSD^12^	512	48.5	28.8	30.3	10.9	31.8	43.5
CornerNet^26^	512	57.8	40.5	45.3	20.8	44.8	56.7
CenterNet^27^	512	62.4	44.9	48.1	25.6	47.4	57.4
YOLOv3^15^	608	57.9	33.0	34.3	18.3	35.4	41.9
ASFF^33^	608	63.0	42.4	47.4	25.5	45.7	52.3
Efficientdet^32^	896	65.0	**45.8**	*49.3*	**26.6**	49.4	59.8
FCOS^28^	800$\times$1024	64.1	44.7	48.4	*27.6*	47.5	55.6
DETR-DC5-R101^30^	800$\times$1333	64.7	44.9	47.7	23.7	49.5	**62.3**
YOLOv4^17^	512	64.9	45.4	48.7	23.0	**50.7**	*62.7*
Ours	512	**65.4**	45.6	**48.9**	25.4	*51.0*	62.0
ViDT-tiny^31^	800$\times$1333	64.5	44.8	48.7	25.9	47.6	62.1
ViDT-base^31^	800$\times$1333	*69.4*	*49.2*	${\varvec{53.1}}$	${{\varvec{30.6}}}$	${{\varvec{52.6}}}$	${{\varvec{66.9}}}$
YOLOv8-S^21^	640	61.8	44.9	–	–	–	–
YOLOv8-L^21^	640	${{\varvec{69.8}}}$	${{\varvec{52.9}}}$	–	–	–	–

The proposed method achieved the best detection results on $AP_{50}$, which refers to the mean average precision (mAP) for intersection over union (IoU) value thresholds equal to 0.5. $AP_{50:95}$, $AP_{75}$, $AP_{S}$, $AP_{M}$ and $AP_{L}$ refer to the average mAP across value of IoU thresholds (i.e., $AP_{50:95}$ refers to the average of 10 mAP across IoU thresholds). Bold-italic, italic and boldfaced values represent the best, second-best, and third-best results in each column, respectively.

**Figure 5:**
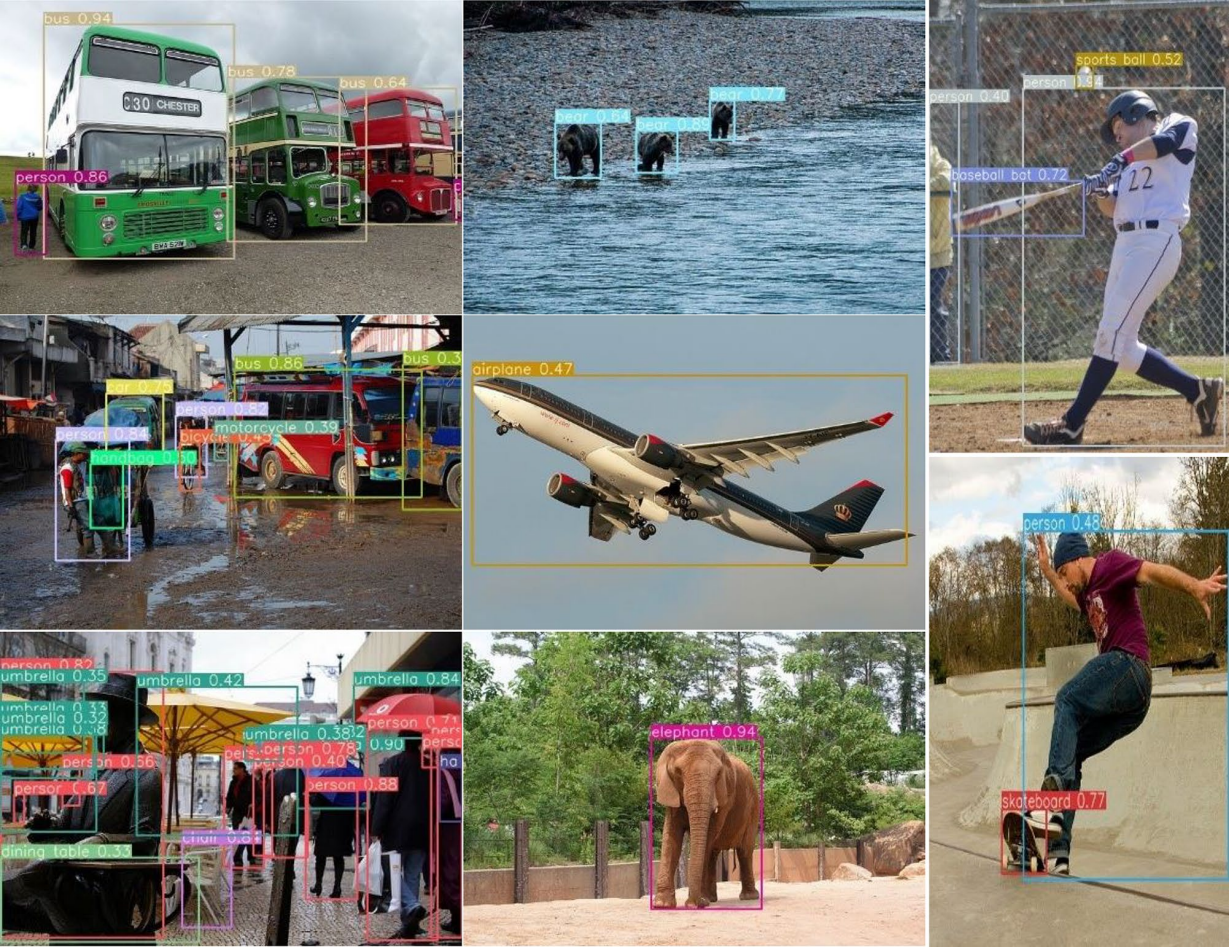
The object detection using KR–AL–YOLO on the COCO testing dataset.

The object detection results of KR–AL–YOLO on the COCO test-val2017 dataset are presented in Tables 1 and 2, as well as Fig. 5. Table 1 provides a comparison of the performance improvements achieved by each component of KR–AL–YOLO, while Table 2 compares the detection accuracy of KR–AL–YOLO with state-of-the-art approaches. The evaluation metric used is consistent with YOLOv4 for all reported results.

Table 1 demonstrates that KR–AL–YOLO, with YOLOv4 as the baseline, achieves a 0.1% increase in average precision (*AP*) and a 0.4% increase in $AP_{50}$ through the addition of keypoint regression alone. Notably, the proposed method exhibits significant improvements in the detection accuracy of small objects. The $AP_S$ of YOLO+AL surpasses that of YOLOv4 by 3.2%. However, the accuracy of detecting large objects is lower compared to YOLOv4 due to the higher sensitivity of the IoU rate to bounding box offsets for small objects. Even slight deviations can have a substantial impact on the overall position of the bounding box for small objects. The angle-loss function imposes a heavier penalty on small targets during model training since deviations lead to larger angles in the small target boxes.

Table 2 presents a comparison between the proposed method and other state-of-the-art object detection algorithms on the COCO test-val2017 dataset. Leveraging the robust baseline of YOLOv4 and employing various training techniques, the proposed detector achieves high detection accuracy, surpassing YOLOv4 and EfficientDet by 0.5% and 0.4% in $AP_{50}$, respectively. Our approach outperforms both the ViDT-tiny method and YOLOv8-S method across multiple metrics. However, compared to the ViDT-base and YOLOv8-L methods, our approach falls slightly behind in various indicators. ViDT-base and YOLOv8-L methods do indeed stand as some of the most advanced object detection techniques at present. The primary reason for this disparity is that our approach is built upon the YOLOv4 framework and lacks the integration of additional optimization techniques to enhance algorithmic performance. Additionally, as seen from Table 2, our approach does not perform very well in detecting small objects compared to the state-of-the-art (SOTA) methods. We believe that certain SOTA methods, specifically designed for optimizing small object detection, such as RSOD^16^, are likely to achieve better results on the COCO dataset when it comes to detecting small objects. The RSOD^16^ method utilizes shallow feature maps to construct a feature pyramid network, enhancing the extraction of fine-grained image features. It integrates an improved spatial pyramid pooling layer into the lateral connections of the feature pyramid network (FPN) to enhance the extraction of local and global image features. By employing adaptive weight allocation, it effectively enhances valuable information while suppressing irrelevant details. Techniques like fine-grained feature pyramid construction and adaptive weight allocation have the potential to significantly improve the accuracy of small object detection, providing valuable insights for our future research endeavors. The detection results of the proposed method on the COCO testing dataset are visualized in Fig. 5.

## Conclusions

In this paper, we present KR–AL–YOLO, an object detection approach that enhances the bounding box regression strategy in YOLOv4. KR–AL–YOLO introduces an keypoint regression strategy and an angle loss to improve the recall rate and overall detection performance. Additionally, a feature fusion method is employed to effectively gather and concatenate multiscale features, facilitating comprehensive object feature learning. Experimental results on the COCO2017 datasets validate the superiority of KR–AL–YOLO over YOLOv4, particularly in the accurate detection of small objects.

## Data Availability

The datasets generated and analysed during the current study are available in COCO2017 (https://cocodataset.org/#download).
